# Advances in multimodality molecular imaging

**DOI:** 10.4103/0971-6203.54844

**Published:** 2009

**Authors:** Habib Zaidi, Rameshwar Prasad

**Affiliations:** Geneva University Hospital, Division of Nuclear Medicine, CH-1211 Geneva, Switzerland

**Keywords:** Instrumentation, multimodality imaging, PET, PET/CT, PET/MR

## Abstract

Multimodality molecular imaging using high resolution positron emission tomography (PET) combined with other modalities is now playing a pivotal role in basic and clinical research. The introduction of combined PET/CT systems in clinical setting has revolutionized the practice of diagnostic imaging. The complementarity between the intrinsically aligned anatomic (CT) and functional or metabolic (PET) information provided in a “one-stop shop” and the possibility to use CT images for attenuation correction of the PET data has been the driving force behind the success of this technology. On the other hand, combining PET with Magnetic Resonance Imaging (MRI) in a single gantry is technically more challenging owing to the strong magnetic fields. Nevertheless, significant progress has been made resulting in the design of few preclinical PET systems and one human prototype dedicated for simultaneous PET/MR brain imaging. This paper discusses recent advances in PET instrumentation and the advantages and challenges of multimodality imaging systems. Future opportunities and the challenges facing the adoption of multimodality imaging instrumentation will also be addressed.

## Introduction

Multimodality molecular imaging is now playing a pivotal role in clinical setting and biomedical research. Modern molecular imaging technologies are deemed to potentially lead to a revolutionary paradigm shift in healthcare and revolutionize clinical practice. Within the spectrum of macroscopic medical imaging, sensitivity ranges from the detection of millimolar to sub-millimolar concentrations of contrast media with computed tomography (CT) and magnetic resonance imaging (MRI), respectively, to picomolar concentrations in single-photon emission computed tomography (SPECT) and positron emission tomography (PET): a 10^8^-10^9^ difference.[1]

**Figure 1:**
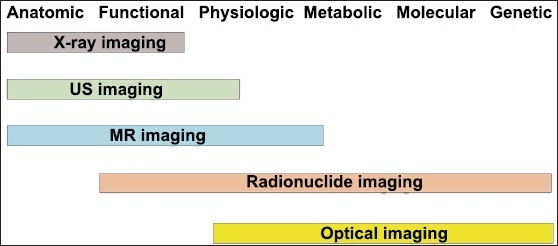
Extent, over the imaging applications, of the most popular medical imaging modalities based on their widespread use

Multimodality imaging has emerged as a technology that utilizes the strengths of different modalities and yields a hybrid imaging platform with benefits superior to those of any of its individual components, considered alone. This imaging trend has been stepped up in recent years due to the emergence of modern molecular biology, advanced biomedical technology and imaging sciences, which requires a highly combined synergistic and visualization approach. This is because molecular imaging, by definition, renders information that can not be provided by conventional radiological imaging, but regardless needs integration of anatomy and function to be fully understood. Figure 1 depicts grossly the extent of each imaging modality based on its widespread use. We focus in this review on the various approaches used in clinical multimodality molecular imaging.

## Trends in PET Instrumentation

The early exploitation of positron decay for medical applications can be said to be the early brain scanning experiments conducted as a result of the pioneering work of Brownell, Sweet, and colleagues at Massachusetts General Hospital who devised the first apparatus based on coincidence detection to localize brain tumors.[2–4] Likewise, most of the first human PET prototypes were developed specifically for functional brain imaging.[5–6] This combined effort of various researchers has led to the development of a functional state-of-the-art PET acquisition and reconstruction technology.[7] The better performance of full-ring systems compared to camera-based dual or triple-headed systems is due to the higher overall system efficiency and count rate capability which provides the statistical optimization of the physical detector resolution and not a higher intrinsic physical detector resolution. This has some important design characteristics since even if both scanner designs provide the same physical spatial resolution as estimated by a point spread function, the full-ring system will produce higher resolution images due to the higher statistics collected per unit imaging time. The geometry of the PET scanner design actually affects to a greater extent the solid angle aperture, which has direct consequences on the resulting sensitivity.

PET is now used in many clinical applications including neurology, psychiatry, cardiology and oncology but honors its success to clinical oncology in which whole-body scanning has a central role in diagnosis, staging, assessment of response to therapy and surgery as well as radiation therapy planning.[8]

On the other hand, the reconstruction algorithm used in PET imaging significantly affects the achieved spatial resolution, image quality and quantitative accuracy. It has been shown that iterative algorithms surmount conventional analytic methods.[9] Important performance parameters of a PET system include sensitivity, spatial, energy, temporal and contrast resolutions, counting rate, dead time, scatter fraction and many other parameters. The sensitivity is the fraction of all coincident 511-keV photon pairs emitted from the object that are recorded by the system, which also is referred to as the coincidence photon detection efficiency. This parameter determines the statistical quality of image data for a particular acquisition time.[10]

**Figure 2:**
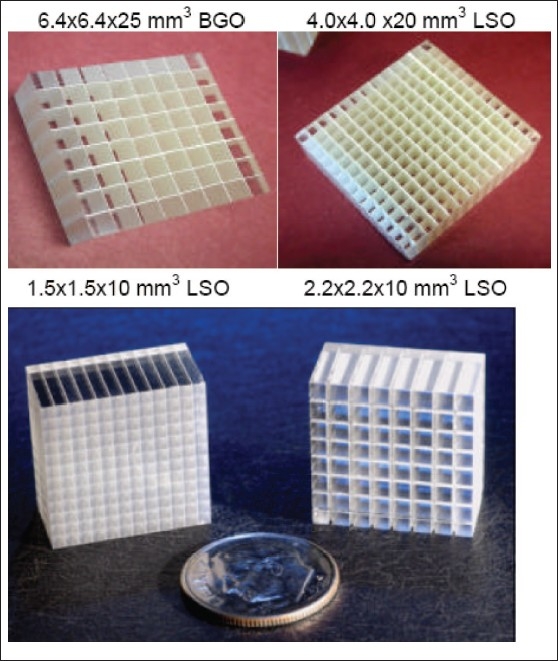
Photographs of different geometrical arrangements of clinical (top) and preclinical (bottom) PET block detector design realized using an assembly of 8×8 BGO crystals of 6.45×6.45×25 mm^3^ each (top left) and 13×13 LSO crystals of 4×4×20 mm^3^ (top right). (Courtesy of Siemens Medical Solutions). Example 8×8 arrays of discrete LSO scintillation crystal pixels used in two successive generations of microPET systems (Courtesy of Siemens Preclinical Solutions, Knoxville, USA). In these last system designs, each array is coupled to a position sensitive photomultiplier tube through fiber-optic coupling

The spatial resolution describes a system's ability to distinguish two closely spaced signals of radioactive concentrations and is important to detect and separate two closely located small sources. Higher spatial resolution can be achieved through the use of smaller detector elements to provide finer sampling of the biodistribution of interest [Figure 2]. Energy resolution is the precision with which one can measure the incoming photon energy. Since detected scattered photons loose energy, a good energy resolution may allow to use a narrow energy window to reduce scatter photon contamination in image data. A narrow energy window also helps to reduce the rate of random photon contamination since many of these photons also undergo scatter. The coincidence time resolution determines how well one can detect whether two coincident photons truly arrive simultaneously. The energy and temporal resolutions are both responsible for the system contrast resolution, which is the ability to differentiate two slightly different radioactivity concentration levels in adjacent targets.

Recent developments of new PET detector modules and scanner designs have followed three main trends:

1. PET systems with depth of of interaction (DOI) information;
2. Designing detector modules and scanners for specific purposes, namely brain, breast, prostate and small-animal imaging. For these applications, detectors with very high spatial resolution are required.
3. Combining and permutating various individual modalities, such as, PET and x-ray CT, MRI or optical tomography to build multimodality imaging units.

New detection technologies that emerged include the use of new cerium (Ce) doped crystals (e.g. LSO, GSO, LYSO, LaBr3) as alternatives to conventional bismuth germanate (BGO) crystals[11] and the use of layered crystals (phoswich detectors) and other schemes for DOI determination, and a renewed interest in old technologies such as time-of-flight (TOF)-PET taking advantage of new developments in scintillator technology. Phoswich detectors consist of two detectors that are assembled in a sandwich-like design, where the difference in decay time of the light is used to estimate depth in the crystal where the interaction occurred. In TOF-PET, the measure of difference of the arrival times of the 511 keV annihilation photons allows restricting the position of positron emission to a subsection (cord) of the coincidence line connecting the two scintillation crystals. This technique was suggested and developed with limited success in the 1980s where the drawbacks associated with the lack of fast scintillators combining excellent timing resolution and high stopping power impeded their wide adoption. With the introduction of new fast scintillator crystals, TOF is now a feasible option which allows to improve the signal-to-noise ratio through incorporation of TOF information.[12]

Image reconstruction in PET began with conventional filtered backprojection methods, including fully 3D reconstruction algorithms which are linear, and computationally fast, but lead in some cases to visible artifacts caused by a combination of low statistics (both in the original emission data with additional possible contributions from normalization, attenuation correction, and randoms correction) and sampling considerations. These methods model the geometry of the scanner with perfect point-like detectors and give the same weight to projection elements containing large numbers of counts as to those containing just a few counts. For these reasons, the trend led to the development and widespread use in clinical setting of iterative reconstruction algorithms that weight the data according to their statistical quality and that accurately model the geometry of the imaging system, including effects such as intercrystal scatter and depth of interaction effects and nonuniform sensitivity along a line of response. These methods can also handle the biasness of corrections for attenuation, scatter and normalization in a statistically optimized way. Iterative algorithms usually result in reconstructed images that have a more optimized tradeoff between signal to noise ratio (SNR) and spatial resolution and in these algorithms streak artifacts, common in filtered backprojection methods (e.g., around high radioactivity uptake areas in ^18^F-FDG studies), are effectively eliminated.[13] The major drawback has been the computational cost of these algorithms. As the consequence of progress, accelerated versions of these algorithms were developed that made their use possible for clinical routine and high end research.

## Special Purpose Dedicated PET Scanners

After the rapid growth and practical utility of whole-body PET scanners, the need to develop organ-and application-specific small field-of-view (FoV) PET scanners was soon realized. PET systems designed for human brain imaging are superior in performance in terms of spatial resolution, sensitivity and SNR which is possible due to proper selection of the design geometry (usually smaller bore and detector crystals), detector assembly and readout electronics as well as optimized data acquisition protocols and image reconstruction algorithms. As an example, Braem *et al*.[14] proposed a novel detector design, which provides full 3-D reconstruction free of parallax errors with excellent spatial resolution over the total detector volume. The key components are a matrix of long scintillator crystals coupled on both ends to HPDs with matched segmentation and integrated readout electronics. The recent development in this field is to extract the axial coordinate from a matrix of long axially oriented crystals, which is based on wavelength shifting (WLS) plastic strips. This method allows building compact 3-D axial gamma detector modules for PET scanners with excellent 3-D spatial, timing and energy resolution while keeping the number of readout channels reasonably low. A voxel resolution of about 10 mm^3^ is claimed.[15]

Early detection of breast cancer is crucial for efficient and effective treatment. Dedicated breast PET imaging, called positron emission mammography (PEM), is a relatively recent technique allowing to obtain images of the breast for detection of radiotracer enhanced tumors. A number of dedicated PEM cameras optimized to image the breast have been proposed or constructed. These cameras restrict the FoV to a single breast, and have higher performance and lower cost than a conventional PET scanner dedicated for whole-body imaging. By placing the detectors close to the breast, the PEM geometry subtends more solid angle around the breast than a conventional PET camera. In addition, annihilation photons emitted in the breast have to pass through at most one attenuation length (~10 cm) of tissue in the PEM geometry, but may have to travel through as much as four attenuation lengths of tissue in a conventional PET camera. These two factors significantly increase the sensitivity (the detected coincident event rate per unit activity in the FoV) in the PEM geometry.[16]

One example of a high resolution PET scanner dedicated for prostate imaging uses an elliptical geometry based on curved detector banks.[17] The distance between detector banks can be adjusted by the user to allow patient access thus accommodating patients of different size and to put the detectors as closely as possible to the prostate gland for maximum sensitivity through optimal solid angle coverage. The detector crystals are inclined in such a way to face the prostate thus allowing to reduce parallax error related spatial resolution degradation in that region.

Another field which is growing rapidly is the development of PET scanners for imaging small-animals, especially rodents (mice and rats). PET's ability to measure biochemical function, rather than structure, can provide crucial insight into the functioning of already existing and new pharmaceuticals, the nature of diseases, or the function of specific genes. As in human imaging, both high detection sensitivity and excellent spatial resolution are priorities for PET imaging system design and are needed to achieve suitable levels of image quality and quantitative accuracy. Thus, different preclinical PET designs have been suggested encompassing conventional small ring radius cylindrical block-detector based design with DOI capability and avalanche photodiodes (APD's) readout, a renewed interest in the 3D high-density avalanche chamber (HIDAC) camera that achieves millimeter-scale spatial resolution along with many other designs. Several high-resolution small animal scanner designs have been or are being developed in both academic and corporate settings, with more than seven such devices being offered commercially.[18] More recently, advanced versions of these technologies have begun to be used across the breadth of modern biomedical research to study non-invasively small laboratory animals in a myriad of experimental settings. The first commercially available microPET system,[19] developed originally at UCLA, consists of a cylindrical arrangement of 2×2×10 mm^3^ LSO crystals readout by short optical fibres to multi-channel PMT's [Figure 2]. The microPET Focus is the next generation microPET system, which uses ~1.5 mm square crystals and achieves a spatial resolution less than 1.3 mm in the center of the FoV using a statistical reconstruction algorithm incorporating accurate system modelling. The yttrium-aluminumperovskite (YAP)-PET system developed by the Universities of Ferrara and Pisa (Italy) comprises four rotating heads spaced 15 cm apart, each with an active area of 4×4 cm^2^, containing a 20×20 array of 2×2×3 mm^3^ optically isolated YAP crystals coupled to PSPMTs, forming a 4-cm transaxial and axial FoV.[20] The reconstructed spatial resolution and absolute photon sensitivity are 1.8 mm FWHM and 1.7% (50 keV threshold) for a centered point source, respectively.

Oxford Positron Systems' (Wadsley Grove, UK) HIDAC PET scanner is a specialized high resolution gas multi-wire proportional chamber (MWPC) imaging system developed at CERN, modified and refined for small-animal imaging.[21] In this position sensitive gas ionization chamber, the annihilation photons are converted by lead cathode plates into electrons, which subsequently are detected and localized by collecting the ionizations generated as they drift and avalanche in the gas. With the widespread availability of commercial preclinical PET systems, small-animal imaging is becoming readily accessible and increasingly popular.

## Advances in Clinical Multimodality Molecular Imaging

Even though the introduction of dedicated dual-modality imaging systems designed specifically and available commercially for clinical practice is relatively recent, the concept of combining anatomical and functional imaging has been recognized for several decades.[22] In late 80's and early 90's, investigators from the University of California, San Francisco, pioneered the development of hybrid SPECT/CT devices which could record both SPECT and x-ray CT data for correlated functional/structural imaging.[23] The system used an array of semiconductor (HPGe) detectors with sufficient energy discrimination and count-rate performance to discriminate x-rays emitted by an internally distributed radiopharmaceutical from x-rays transmitted through the body from an external x-ray source. Because the HPGe detector implemented in the first two prototypes was expensive and impractical for clinical use, the same group next implemented a SPECT/CT scanner for patient studies by sitting a GE 9800 Quick CT scanner in tandem with a GE 400 XR/T SPECT system.

The development of combined PET and CT scanners in the same gantry followed the same trend in the latter part of the 90s by investigators from the University of Pittsburgh[24] and has revolutionized the practice of clinical PET. Co-registered PET and CT data provides regions of increased ^18^F-FDG accumulation on the PET image to be directly correlated with their anatomic locations on the CT scan, thus improving the sensitivity and specificity of PET for lesion detection and increasing the accuracy of target volume delineation. The combination of PET and CT has important secondary benefits as well. The CT scan, with appropriate consideration for differences between the spectrum of x-ray energies produced in CT and the monoenergetic 511-keV photons detected in PET and many other physical factors, can be used to correct for photon attenuation in PET,[25] thus eliminating the need for PET transmission sources and scans. The CT scan also has the potential to be used to estimate the Compton scatter magnitude and spatial distribution in the PET scan and to correct for partial volume errors. The advent of combined PET/CT units is a prominent example of an advance in molecular imaging technology that offers the opportunity to modernize the practice of clinical oncology by improving lesion localization and facilitating treatment planning for radiation therapy. Joint efforts between industry and academia is expected to drive fast and innovative improvements of the available technologies, not only the PET subsystem for the combined modality, but also the CT part which is being equipped with faster acquisition boards and designed to accomodate larger volume coverage using multislice technology and optimized radiation dose reduction schemes.

Following the success and wide clinical adoption of PET-CT scanners, integrated SPECT-CT have also been developed and put into clinical practice by the major vendors. The superior diagnostic quality and the much shorter acquisition times achieved by these CT scanners offer significant advantages. Most SPECT/CT systems incorporate a dual-headed SPECT camera coupled to a multi-slice (2, 6, 16 or 64 slice) diagnostic CT scanner whereas current PET/CT systems incorporate a full-ring PET scanner equipped with dedicated detectors allowing either 2D/3D or only 3D PET imaging and a CT scanner having up to 64-slice capability.

**Figure 3:**
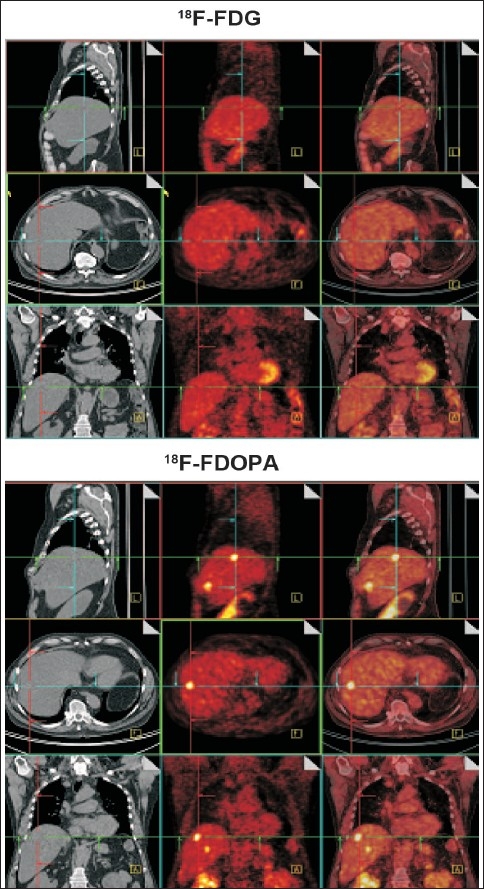
Illustration of a clinical PET/CT study showing the limitations of ^18^F-FDG for the detection of hepatic metastases whereas two metastases were clearly visible on the ^18^F-FDopa study

The wide adoption of FDG (molecule of the century) and a multitude of novel radiotracers have clearly demonstrated the enormous potential of PET-CT as an emerging discipline in the field of molecular imaging. It can arguably be stated that FDG-PET, as a single modality, has made an everlasting impact on the specialty of nuclear medicine. In fact, it has rejuvenated the field and has changed its image in the medical community. However, FDG-PET/CT has limited impact in many malignancies presenting with low FDG avidity, e.g. prostate cancer, hepatic metastases …etc, where more specific tracers should be used [Figure 3].

Despite the success and popularity of PET-CT and more recently of SPECT-CT, there are some shortcomings in the use of CT as the combined anatomical imaging modality. Firstly, CT adds radiation dose to the overall examination, particularly if used in a full diagnostic role with contrast enhancement. Second, CT provides relatively poor soft tissue contrast in the absence of oral and intravenous iodinated contrast, particularly if low dose acquisition protocols are utilized to minimize radiation exposure. These two theoretical limitations do not apply to MRI, which does not involve ionizing radiation and provides soft tissue imaging with high spatial resolution and superior contrast compared to CT. MRI can also provide more advanced ‘functional’ techniques such as perfusion and diffusion imaging as well as spectroscopy, which may be additive to functional information obtained by PET. Furthermore, the high sensitivity of PET may also complement the poor signal strength inherent in current functional MRI imaging. The combination of PET and MRI into a single scanner may therefore be the pioneer hybrid imaging modality, combining the metabolic and molecular information of PET with the excellent anatomical detail of MRI, while offering new potential applications with respect to functional MRI technology.

There have been several recent publications on the value of fused or co-registered PET-MR images in preclinical and clinical practice.[26] There are, however, a number of technical problems that need to be overcome before a clinical hybrid PET-MR scanner can become a reality. Both MRI and PET have the potential to affect each other's performance in their current form. One of the main problems is that photomultiplier tubes, a fundamental component of current PET detectors, will not function in a ‘magnet’ as the high magnetic field causes electrons to deviate from their original trajectory, resulting in loss of gain.[27] A small prototype PET-MRI scanner has been developed using long optical fibres to transport light from the detector to photomultiplier tubes situated in a low field region.[27] The potential of using novel readout technologies insensitive to magnetic fields, including APDs and Geiger-mode avalanche photodiodes (G-APDs) has been and still is being explored for further development. APD-based technology has been successfully implemented by one vendor of small-animal PET scanners[28] and used as building block in many preclinical PET/MR systems.[29–31] G-APDs, small finely pixelated APDs operated in Geiger mode, have a tremendous potential for further improvement and exploration.

The first prototype human PET insert, the BrainPET scanner,[32,33] was designed in 2005 within a collaborative effort between Siemens Medical Solutions (Erlangen, Germany) and the University of Tuebingen, Germany using photodetectors insensitive to magnetic fields (APDs instead of PMTs) and non-magnetic detector and front-end electronic materials to operate within a clinical MRI system. The BrainPET was designed to operate in the frequency range of interest for MRI at 3T, allowing perfect matching with the most sophisticated MR brain sequences that can be performed at this magnetic field strength. The first patient images were shown late in 2006[34] and the system is currently undergoing a detailed evaluation of mutual interference between the two imaging modalities and is being used comprehensively to assess its potential using normal subjects and clinical studies.[35]

## Summary

An overview of current state-of-the art developments in PET instrumentation and emerging dual-modality imaging platforms is provided in this brief review. It should be emphasized that many different design paths have been and continue to be pursued in both academic and corporate settings, which offer different compromises in terms of performance and versatility but in most cases improve the clinical workflow efficiency. It is still uncertain which designs will be incorporated into future clinical systems, but it is certain that technological advances will continue and will enable new multimodality molecular imaging capabilities including PET as an essential component of those technologies. More compact and cost-effective designs of multimodality systems are being explored using a rail-withsliding-bed approach where a sliding CT bed is placed on a track in the floor and linked to a flexible SPECT camera.[36] Similar approaches can be used for integrated PET/MR instrumentation. Various other rail-based, docking and click-over approaches for anatomolecular imaging fusion are also being considered where the possibilities are limited only by the imagination and creativity of researchers.[37] In any case, it is the power of molecular imaging using highly specific radiotracers that are central and not the number of slices of the CT sub-system when considering the example of combined PET/CT.
